# Proximal femoral reconstruction with modular megaprostheses in non-oncological patients

**DOI:** 10.1007/s00264-021-05080-8

**Published:** 2021-07-14

**Authors:** Kevin Döring, Klemens Vertesich, Luca Martelanz, Kevin Staats, Christoph Böhler, Christian Hipfl, Reinhard Windhager, Stephan Puchner

**Affiliations:** Division of Orthopaedics, Department of Orthopaedics and Trauma Surgery, Medical University of Vienna, Vienna, Austria

**Keywords:** Proximal femoral replacement, Megaprosthesis, Periprosthetic joint infection, Aseptic loosening, Periprosthetic fracture, Prosthesis dislocation

## Abstract

**Introduction:**

Multiple revision hip arthroplasties and critical trauma might cause severe bone loss that requires proximal femoral replacement (PFR). The aim of this retrospective study was to analyse complication- and revision-free survivals of patients who received modular megaprostheses in an attempt to reconstruct massive non-neoplastic bone defects of the proximal femur.

Questions/purposes

(1) What were general complication rates and revision-free survivals following PFR? (2) What is the incidence of complication specific survivals? (3) What were risk factors leading to a diminished PFR survival?

**Materials and methods:**

Twenty-eight patients with sufficient follow-up after receiving a modular proximal femoral megaprosthesis were identified. The indications for PFR included prosthetic joint infection (PJI), periprosthetic fracture, aseptic loosening, non-union and critical femoral fracture. Complications were grouped according to the ISOLS-classification of segmental endoprosthetic failure by Henderson et al.

**Results:**

Overall, the complication-free survival was 64.3% at one year, 43.2% at five years and 38.4% at ten years, with 16 patients (57%) suffering at least one complication. Complications were dislocation in eight patients (29%), PJI in 6 patients (21%), periprosthetic fracture in five patients (18%), and aseptic loosening in six patients (21%). Prosthesis stem cementation showed a lower risk for revision in a cox proportional hazard model (95% CI 0.04–0.93, HR 0.2, p = 0.04).

**Conclusion:**

PFR with modular megaprostheses represents a viable last resort treatment with high complication rates for patients with severe proximal femoral bone loss due to failed arthroplasty or critical fractures. In revision arthroplasty settings, PFR cementation should be advocated in cases of impaired bone quality.

## Introduction

With an ever-increasing life expectancy and a growing number of arthroplasties performed annually, it comes as no surprise that the incidence of revision total hip arthroplasties (THA) is expected to increase in the foreseeable future [1]. Post-THA stress shielding, bone remodelling and prosthetic wear debris may lead to severe femoral bone loss, which in turn may induce aseptic loosening, migration and periprosthetic fracture [2–9]. Severe femoral bone loss may also be a result of many other arthroplasty-related and -unrelated factors, such as periprosthetic joint infection (PJI), osteoporosis, failed osteosynthesis, trauma, non-union, multiple arthroplasty revisions, and extensive primary bone tumour or metastases resection [4, 7–14].

Reconstruction of major segmental long bone defects is a demanding surgical procedure that poses multiple challenges for the treating orthopaedic surgeon [15]. Treatment options for patients with massive bone loss are limited to allograft-prosthesis composites, or so-called bioimplants, and megaprostheses for proximal femoral reconstruction (PFR) [11]. Other treatment options for patients with lesser bone loss around the proximal femur include impaction allografting, long cemented or press-fit femoral stems, and resection arthroplasty [7–9, 11, 12, 16].

Megaprostheses, also known as tumour endoprostheses, were originally developed for limb salvage surgery after radical excision of primary malignant bone tumours, such as osteosarcoma and chondrosarcoma, primary benign aggressive bone tumours such as giant cell tumour, and destructive metastatic lesions such as cancer metastases [17–19]. At the Medical University of Vienna, tumour endoprostheses have been in use since the 1970s due to aforementioned indications. [20] Megaprostheses have since found application in non-oncological orthopaedics and trauma surgery. A near absolute shift from proto-implants in customized monoblock design to modular megaprostheses towards the end of the last century meant that megaprostheses could intra-operatively be assembled from separate components, to optimally fit specific bone defects. Moreover, unexpected extensive bone deficiencies could now be tackled intra-operatively without manufacturing delay and postponement of surgery. Further refinements in megaprosthetic design and materials improved the functional outcome as well as complication-free survival [13].

The aim of this study was to investigate complication- and revision-free survivals of modular megaprostheses in patients undergoing PFR due to end-stage revision of standard hip endoprostheses or critical trauma.

### Questions/purposes

The following questions were therefore asked: (1) What were general complication rates and revision free survivals following PFR? (2) What is the incidence of complication specific survivals? (3) What were risk factors leading to a diminished PFR survival?

## Materials and methods

### Data collection

The research proposal for this study was accepted by the Ethics Committee of the Medical University of Vienna. A retrospective data analysis in the patient administration system of the Medical University of Vienna identified 40 patients who had undergone PFR due to non-oncological indications from January 1983 to May 2015. One non-surgical related death was reported within the first post-operative year. Altogether, 12 patients were lost to follow-up by defining a minimum follow-up period of 12 months. Thus, 28 patients were left for statistical analysis (Table 1).

**Table 1:** Demographic statistics of patients included and lost to followup. Time comparison in years if not further specified

Parameter	Included (n = 28)	Lost to followup (n = 12)	p
Mean Age at PFR surgery	67 (min = 42, max = 88, SD 13)	78 (min = 51, max = 95, SD 12)	**0.015**^** T**^
Followup after surgery	94 months (min = 12, max = 294, SD 70)	2 months (min = 0.2, max = 6, SD 2)	**0.001**^** T**^
Sex			
Male/Female	6/22	3/9	0.804^#^
Indication for PFR			
Aseptic loosening	5	3	0.605^#^
Periprosthetic fracture	10	5	0.722^#^
Infection/septic loosening	11	3	0.385^#^
Femoral non-union	1	1	0.527^#^
Recurrent luxation	1	0	0.507^#^

Differences between groups tested via

^T^ = T-test

^#^ = Chi-square-test

SD = standard deviation

Bold = Statistically significant results (*p* < 0.05)

### Surgical technique and post-operative care

Median age of patients at the time of megaprosthesis implantation was 67 (range: 42–88) years. PFR was performed in 22 female patients (79%) with a median age of 69 (range: 42–88) years and 6 male patients (21%) with a median age of 57 (range: 43–82) years (Table 2).

**Table 2:** Revision-free survival of different parameters

Parameter	Patients (n = 28)	p
Mean Age at PFR surgery	67 (min = 42, max = 88, SD 13)	0.43^ T^
Number of previous surgeries	2.9 (min = 0, max = 8, SD 1.9)	0.24^ T^
Time from first prosthesis to Megaprosthesis	10 (min = 0, max = 52, SD 10)	0.97^ T^
Mean hospital stay	20 days (min = 7, max = 68, SD 13)	0.61^ T^
Followup after surgery	94 months (min = 12, max = 294, SD 70)	0.86^ T^
Sex		
Male/Female	6/22	0.88^*^
Indication for PFR		
Aseptic loosening	5	0.76^*^
Periprosthetic fracture	10	0.82^*^
Infection/septic loosening	11	0.91^*^
Femoral non-union	1	0.37^*^
Recurrent luxation	1	0.29^*^
Prosthesis type		
KMFTR	15	0.83^*^
HMRS	3	0.41^*^
GMRS	10	0.70^*^
Operative procedure		
Resection length	17 cm (min = 8, max = 30, SD 5)	0.32^ T^
Prosthesis cementation	12	0.04^*^
Trochanter cerclage wires	13	0.58^*^
LARS band augmentation	7	0.47^*^
Postoperative immobilization		
Hip to leg cast or orthosis	18	0.24^*^
Restricted weight bearing with crutches only	8	0.39^*^
No data	2	0.14^*^

Time comparison in years if not further specified

^T^ = T-test

^*^ = log-rank test

Bold = Statistically significant results (*p* < 0.05)

Indications for primary surgical treatment were hip osteoarthritis in 15 patients (54%), femoral neck fracture in 11 patients (39%), chronic polyarthritis and severe infection with osteomyelitis in one patient (4%) each. Surgical procedures subsequently carried out were total hip arthroplasty in 24 cases (86%), osteosynthesis of the proximal femur by either plating or hip screws in two cases (7%), and external fixation in one case (4%). Reconstruction of the proximal femur with a modular megaprosthesis was the indicated primary surgical procedure in one patient (4%) with a problematic Vancouver 3B proximal femoral fracture.

Patients had undergone a median of three (range: 0–8) operations prior to PFR. PFR was performed after PJI in 11 patients (39%), due to periprosthetic fracture in ten patients (36%, patient case 1), aseptic loosening in five patients (18%, patient case 2), recurrent luxation and bone loss in one patient (4%), and, as previously stated, because of the complexity and severity of a proximal femoral fracture as primary implant in one patient (4%). No patient was treated with bilateral PFR.

Reconstruction of the proximal femur was performed using the *Kotz Modular Femur and Tibia Reconstruction System* (*KMFTR*; Howmedica GmbH, Kiel, Germany) or its successor, the *Howmedica Modular Reconstruction System* (*HMRS*; Howmedica Osteonics Corporation, Mahwah, NJ, USA) in 18 patients (64%). In ten patients (36%) the *Global Modular Replacement System* (*GMRS*; Stryker Corporation, Kalamazoo, MI, USA) was implanted.

Patients received treatment with antibiotics intravenously for five to ten post-operative days. Protective weight bearing was performed for six weeks. Sixteen patients were put on post-operative immobilization with hip to leg casts, two patients received hip to leg orthoses, eight patients did not receive any orthosis and in two patients treated in the 1980s, post-operative mobilization was not documented. During their hospital stay (median: 16.5 days, min = 7, max = 68) patients were daily assisted in walking and muscle strength training using a standardized protocol.

### Follow-up examination

Patients were usually invited for a follow-up examination six weeks, six months, 12 months and annually thereafter post-surgery. Median follow-up was 7.3 (range: 1–25) years. Follow-up examination was performed in our outpatient clinic by clinical joint assessment and radiographic imaging. Complications of megaprostheses were classified according to a previously published failure mode classification for tumour endoprostheses into type I or soft-tissue failure; type II or aseptic loosening; type III or structural failure, including periprosthetic fracture and breaking of megaprosthetic components; and type IV or PJI [21]. Failure of muscle and motion function and dislocations of the artificial hip were also classified as type I complications, while the inapt type V complications or tumour progression were omitted entirely.

### Statistical analysis

Descriptive statistics were used to detect frequencies, medians and ranges of megaprosthetic complications. Differences between medians were tested by independent *t*-test for continuous variables or Mann–Whitney *U* Test for nonparametric data. Complication- and revision-free survival was detected using Kaplan–Meier plots. By using a Cox proportional hazard model potentially influencing factors on survival were assessed. Statistical significance was determined by a *p*-value < 0.05. Statistics were performed using *IBM SPSS Statistics 26* (*International Business Machines Corporation*, *IBM*; Armonk, NY, USA) software.

## Results

### General complication and revision-free survivals rates

Overall, 16 patients (57%) suffered from at least one complication after a median period of eight (range: 0.3–81) months. 11 patients (39%) suffered from multiple complications. 15 patients (54%) had to undergo at least one revision surgery after a median period of 12 (range: 0.3–113) months, whereas one patient was treated with one-time closed reduction.

**Fig. 1:**
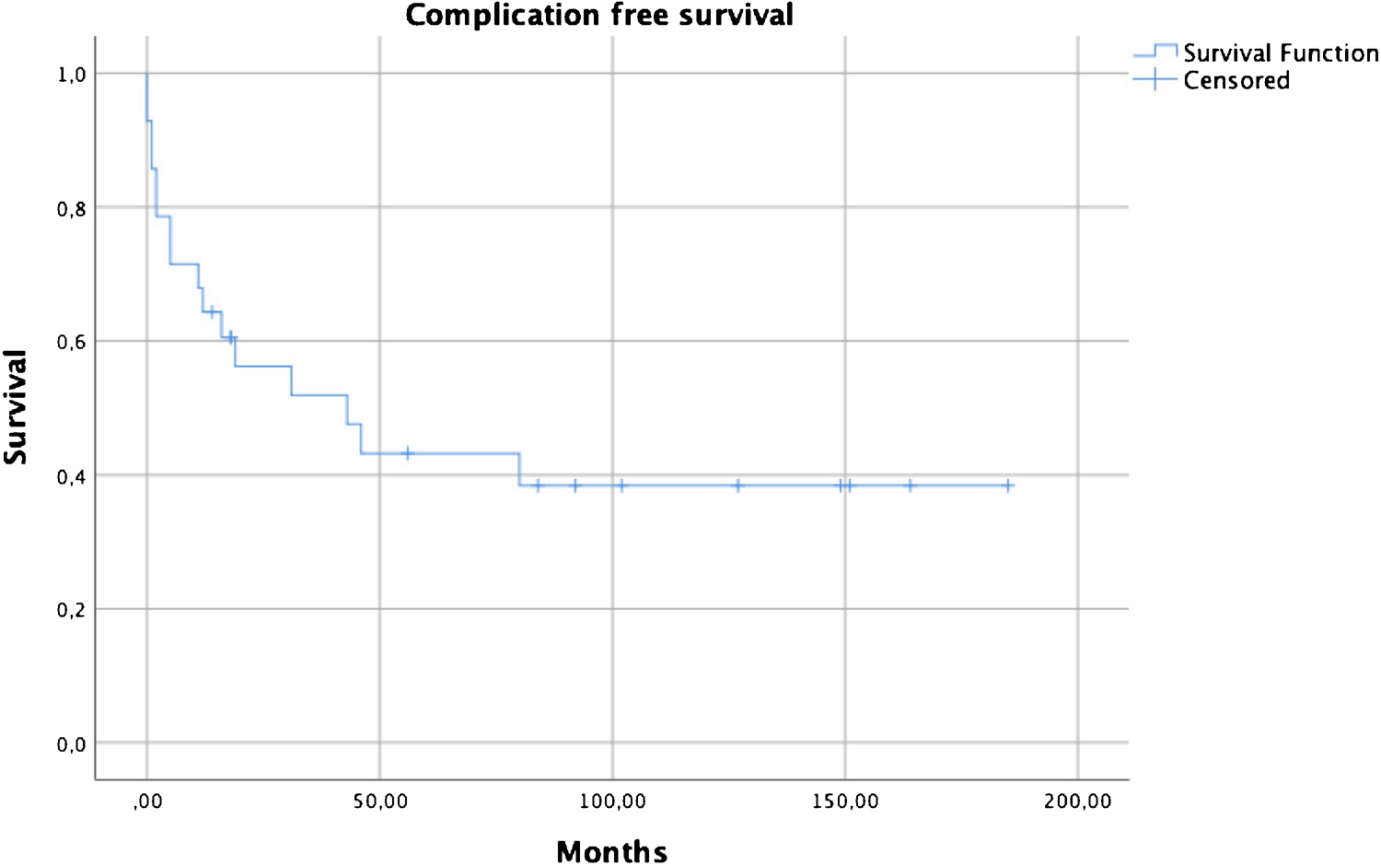
Complication free survival

**Fig. 2:**
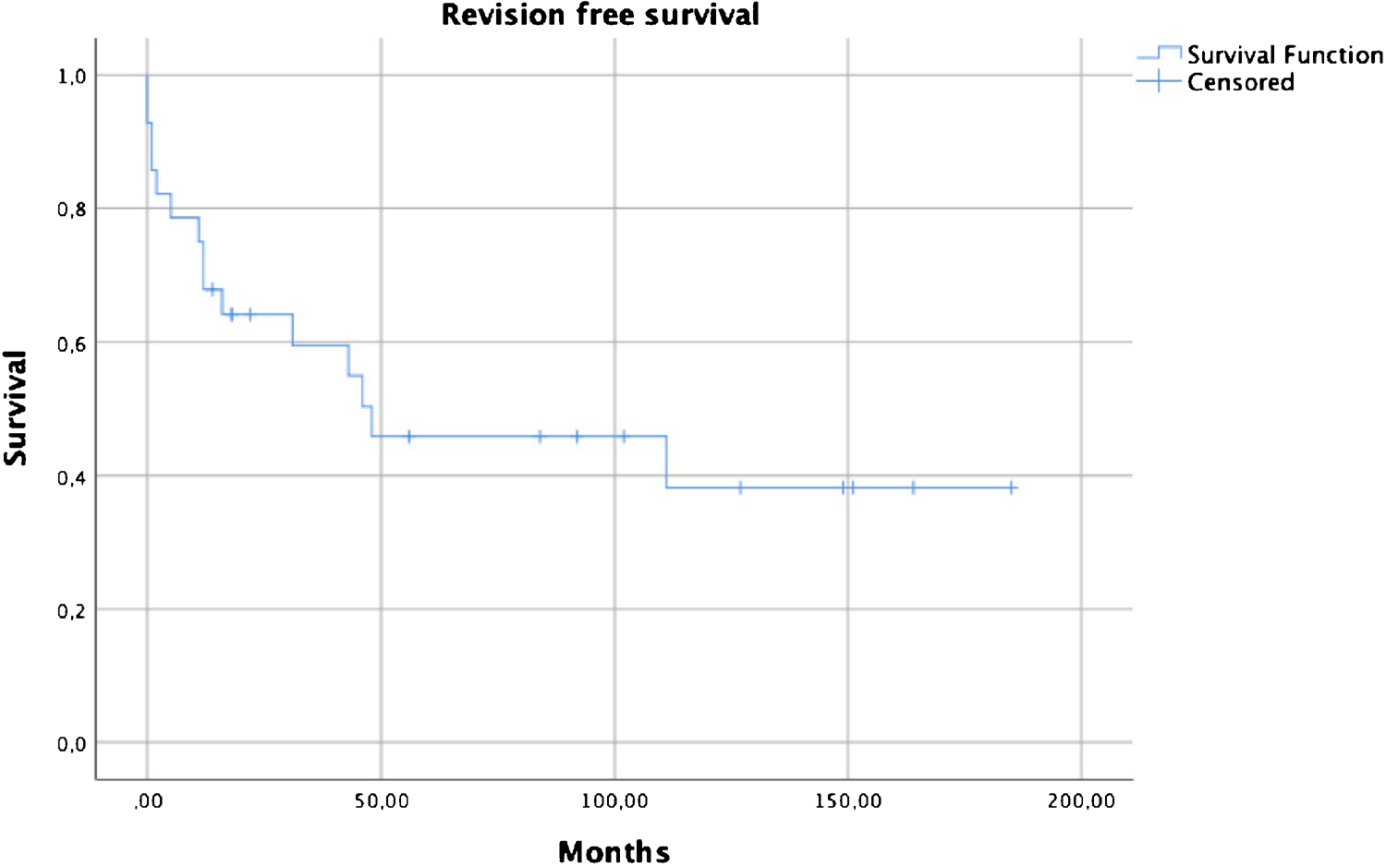
Revision free survival

Overall, the complication-free survival was 64.3% at one year, 43.2% at five years and 38.4% at ten years (Fig. 1); while the revision-free survival was 67.9% at one year, 45.8% at five years and 38.1 at ten years (Fig. 2). There was no statistical difference between general KMFTR/HMRS and GMRS revision free survival (p = 0.70) and complication free survival (p = 0.94).

### Complication-specific implant survival rates

Type I complications occurred in eight patients (29%). All of these patients suffered from dislocation of the modular megaprosthesis. Complication-free survival for type I complications was 77.2% at one year and 64.6% at five and ten years. Three of these patients were solely treated by closed reduction, while five of the patients were treated by at least one open reduction. Three patients (%) suffered from recurrent luxation of the artificial hip. One patient (4%) suffered from re-dislocation after closed reduction of the hip and two patients (7%) suffered from re-dislocation after open reduction of the hip. In seven patients, an artificial band augmentation (LARS — Ligament Advanced Reinforcement System) was used as protection against dislocation. Trochanter refixation with cable wires or sutures was performed in 13 patients, which resulted in 17 patients treated with either trochanter refixation and/or implantation of a LARS band in primary surgery. Both trochanter refixation and usage of LARS bands did not yield any protective effects concerning hip dislocation in statistical analysis (Table 3). In this study, implantation of a LARS band did not lead to a higher rate of periprosthetic joint infection.

**Table 3:** Dislocation analysis

Parameter	n Luxations (Patients)	P^#^	RR
Liner			
Standard liner	4 (16)	0.63	0.8
Elevated liner	3 (5)	0.09	1.4
No information	1 (2)	0.49	1.1
Bipolar head	0 (4)	0.17	-
Dual mobility liner	0 (1)	0.52	-
Trochanter fixation			
Cerclage wires	5 (13)	0.28	1.8
LARS band	4 (7)	0.053	1.7
Trochanteric ETA	0 (1)	0.52	-

^#^, Chi-square test; *RR*, relative risk

Type II complications or aseptic loosening occurred in five patients (18%). Three of these patients presented with loosening of the femoral megaprosthetic component, while two other patients presented with acetabular cup loosening. Revision-free survival for this complication was 87.8% at one year and 74.7% at both five and ten years. Subsequent examinations revealed that two of these patients suffered from recurrent aseptic loosening of the acetabular cup, while two other patients additionally presented with type IV complications.

Type III complications or structural failure occurred in five patients (18%). In all of these cases, the patients suffered from periprosthetic femur fracture. Megaprostheses were exchanged in two patients (7%), while two other patients (7%) underwent total femur reconstruction due to the complexity and severity of the fractures. One patient received a plate osteosynthesis of the periprosthetic fracture. Patients had cemented PFR in two cases, while three other cases received press-fit PFR prior to periprosthetic fracture. Revision-free survival for type III complications was 88.7% at one year and 74.4% at five and ten years.

Type IV complications or PJI occurred in six patients (21%), whereas five patients already had infections of the primary implant prior to undergoing PFR. Hence, only one patient (4%) newly developed PJI. Therapeutic solutions for the treatment of PJI ensued PFR replantation in four cases, while one patient (4%) received a system change to a total femur reconstruction, and one patient (4%) needed hip disarticulation. Revision-free survival for all patients with type IV complications was 83.6% at one year, 76% at five years and 63.3% at ten years. In patients with no infection prior to megaprosthesis implantation, revision free survival for type IV complications was 92.3% after one, five and ten years, whereas for patients with previous infection it was 72.7% after one year, 60.6% after five years and 40.4% after ten years.

Regarding reasons of initial prosthesis failure, no statistical differences between GMRS and KMFTR/HMRS prostheses were found.

### Risk factors for PFR failure

**Fig. 3:**
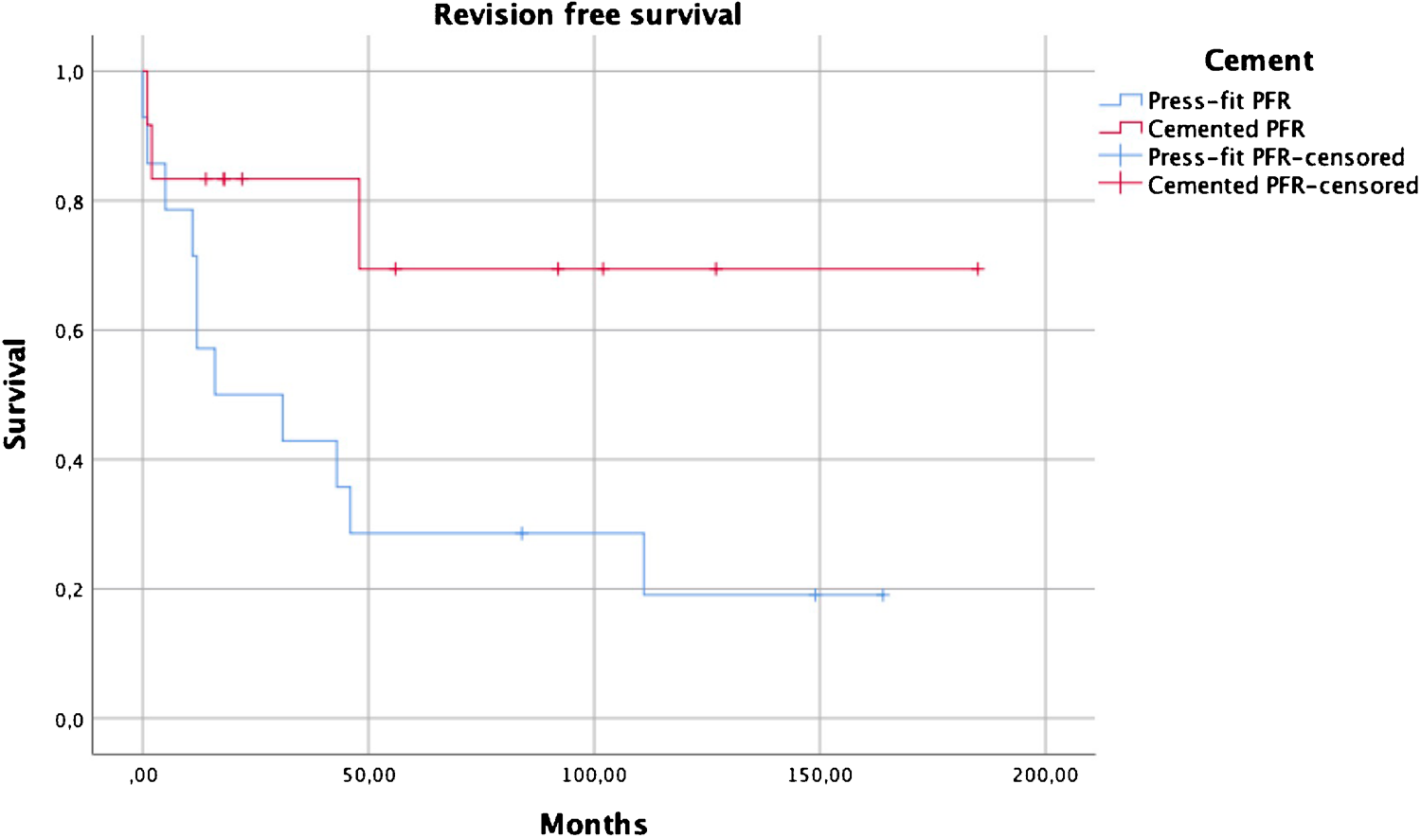
Revision free survival comparing cemented PFR to press-fit PFR (p = 0.04)

**Fig. 4:**
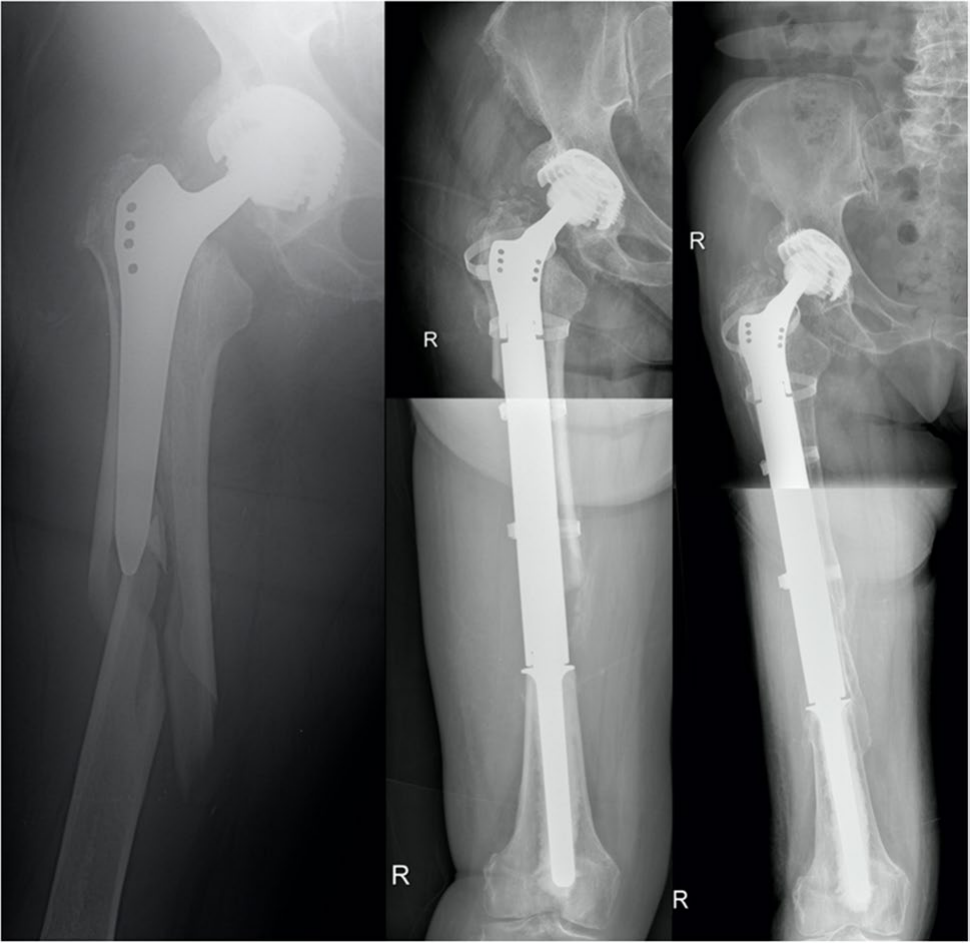
**Left:** Patient 1 suffered from a periprosthetic fracture type Vancouver 3B, which was answered by implantation of a cemented proximal femur GMRS. **Middle:** The proximal femoral shaft was preserved and tied to the prosthesis with cerclages. **Right:** Nine years followup

**Fig. 5:**
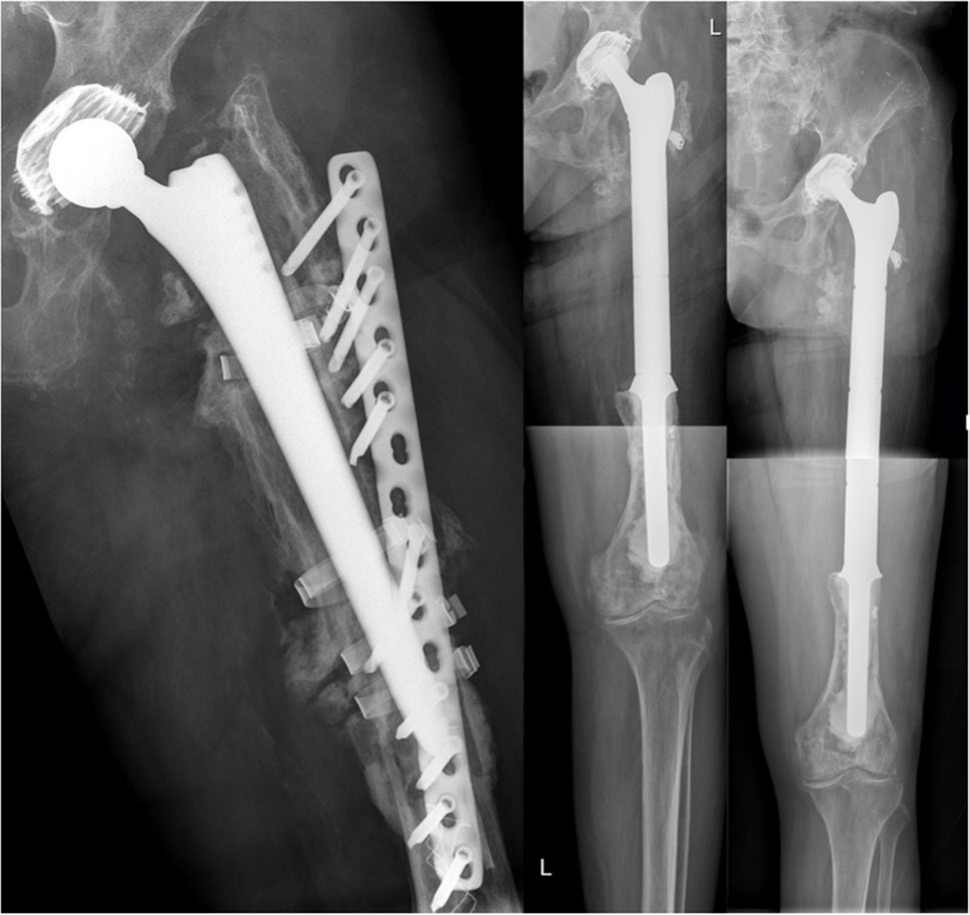
Patient 2 received primary THA due to coxarthrosis in 1995. The patient suffered from recurring dislocations of her artificial hip, which culminated in aseptic loosening of the femoral shaft. The complication was addressed by femoral shaft replantation and femoral head change. In the post-operative course, the patient suffered from a first periprosthetic fracture, which was answered with cerclage wires, and a second periprosthetic fracture which needed the implantation of a 14-hole plate in 2008. **Left:** The patient was admitted to our department with aseptic loosening of the plate in 2009, which led to implantation of a cemented proximal femur GMRS **(Middle)**. **Right:** One year followup

**Fig. 6:**
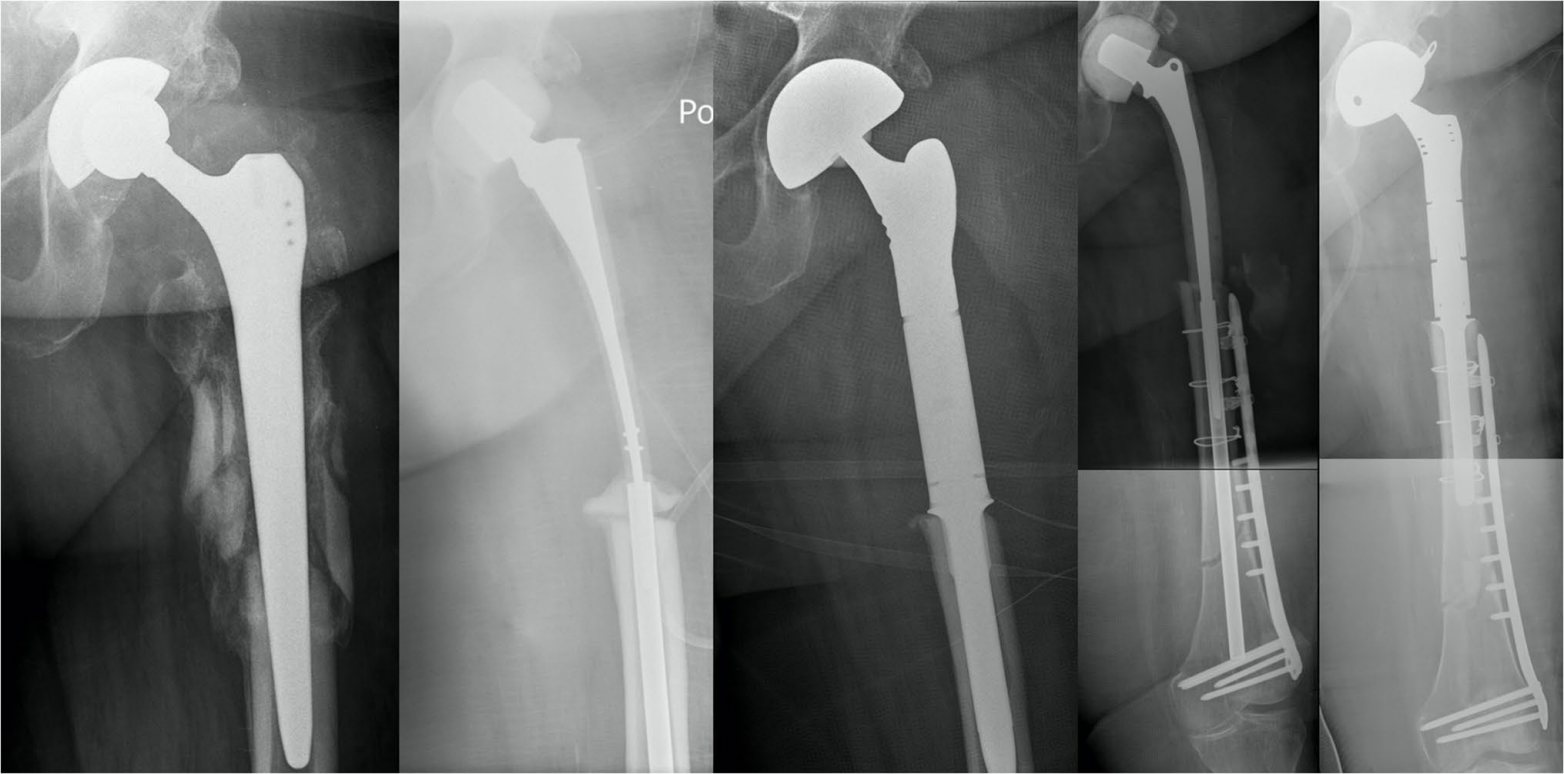
**Left:** Patient 3 had primary THA due to coxarthrosis in 1995 and suffered from Staphylococcus aureus PJI after revision because of periprosthetic fracture prior to admission at our institution in 2013. **Middle left:** Planned two-stage revision with spacer implantation was performed before PFR replantation (**Middle**) after 11 weeks. **Middle right:** PFR explantation was indicated because of recurring PJI one year after index surgery, which led to spacer implantation and osteosynthesis due to intraoperative periprosthetic fracture. **Right:** Revision PFR with a dual mobility cup was performed after 4 months

Concerning revision free survival, implant cementation showed superior results in comparison to press-fit fixation (p = 0.04) in a log rank test (Fig. 3), with no case of aseptic loosening reported after implant cementation. Subsequent estimations of the effects of covariates (age below 60 at surgery, infection prior to megaprosthesis implantation and sex) with the Cox proportional hazard model revealed a lower risk for revision in cemented stem implantations (95% CI 0.04–0.93, HR 0.2, p = 0.04). (Figs. 4, 5, 6)

## Discussion

In non-oncologic settings, PFR is regarded as a salvage option for patients with extreme bone loss, once reconstruction with revision stems is no longer feasible. In our retrospective study that included 28 patients with modular megaprostheses, reconstruction of the proximal femur was indicated in cases of PJI, fracture, aseptic loosening, and fracture non-union.

We identified a couple of limitations in this study, one of them being the remote number of patients whose data were analysed. We would like to think that a larger sample size would not only improve accuracy of the presented findings, but also help identify rare complications of PFR, which could not be detected in this study population, but have been addressed in other scientific works [22, 23]. In order to increase the sample size while keeping the narrowly defined inclusion criteria unchangeable, we would, however, have to undertake a thorough investigation into even older medical records at multiple health care institutions. With an inclusion period of 32 years, not only PFR implants but also knowledge concerning complications and therapy strategies evolved. However, the majority of existent literature on the topic adopted sample sizes that were smaller than the one used in this study [16]. Furthermore, the retrospective nature of this study impeded the collection of additional data and further clinical testing. Detailed pre-operative examination and functional scores were often impracticable or unavailable. Because of patient admission to our clinic after primary THA due to complications, medical records of primary implants were sometimes missing, leading to missing data.

### General complication and revision-free survival rates

**Fig. 7:**
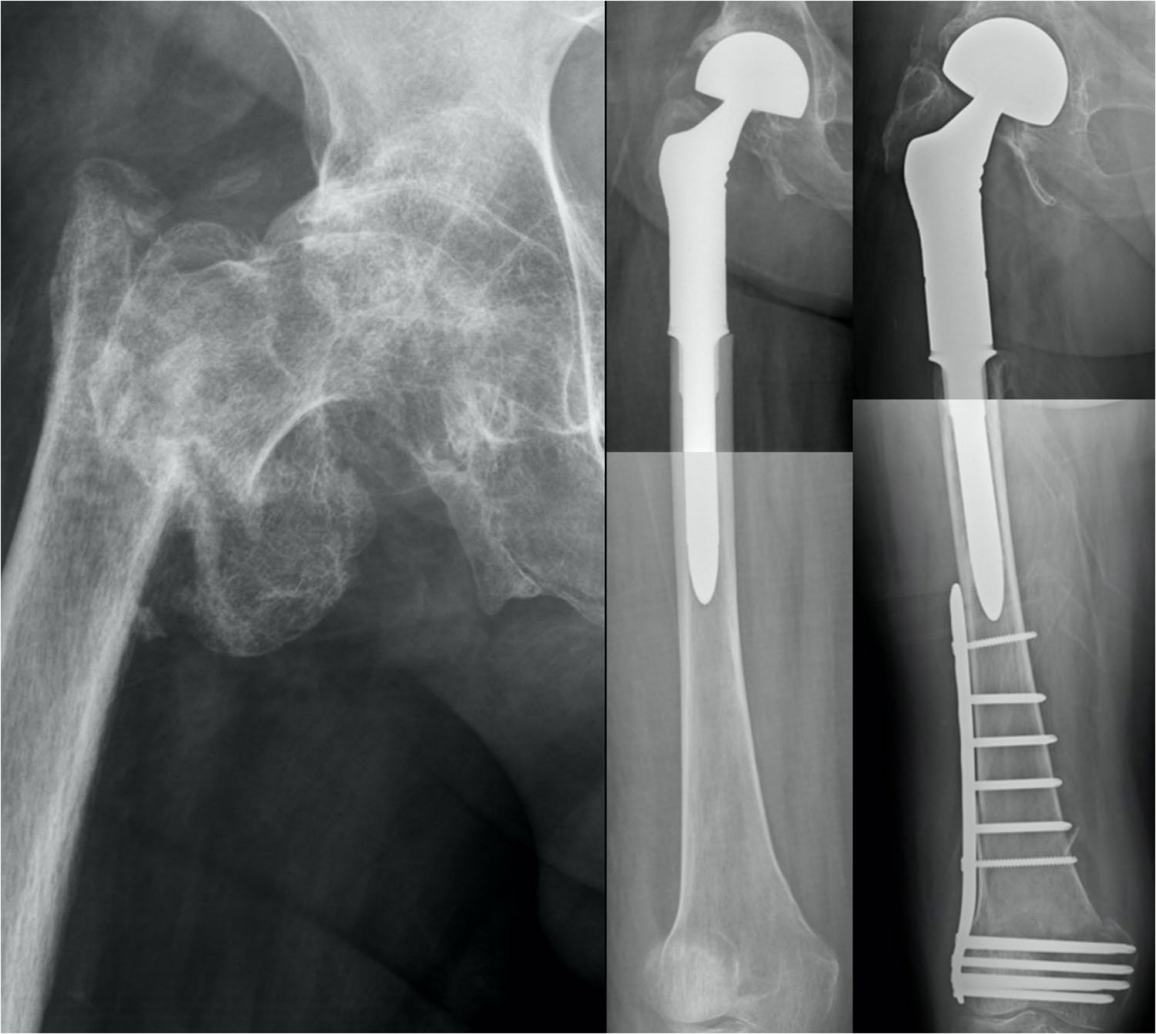
**Left:** Patient 4 suffered from proximal femoral fracture after a fall, which led to PFR implantation **(Middle)**. **Right:** Seven years after surgery, the patient suffered from a supracondylar femur fracture, which was surgically addressed with plate osteosynthesis

The overall complication- and revision-free survival determined in our patient collective were 64.3% and 67.9% at one year, 43.2% and 45.8% at five years, and 38.4% and 38.1% at ten years, respectively. The main difference between our results and those published in previous retrospective case series on non-oncological PFR seems to be the drop in revision-free survival at one year due to dislocation treated with open reduction and aseptic loosening treated with PFR cementation, considering the fact that other studies have described implant survival of 87 to 97% at one year and 32 to 95% at five years [8, 10, 24] (Table 4). In an effort to explain these results, we found a low average patient age (median 67 years) and a high number of patients treated for septic hip conditions (n = 11, 39%) in comparison to other studies. Our prosthetic survival rate with revision as end point was expectedly lower than that of modular megaprostheses used after oncologic resection (94.9% and 90.7% at one and five years, respectively) [19] and the revision rate identified was much higher in comparison to THA in literature (1.31% and 3.25% at one and five years, respectively) [25]. An explanation for higher revision rates in patients with PFR after THA or fracture in comparison to patients receiving PFR due to oncologic resections might be found in different patient populations, as patients treated with PFR for non-oncologic reasons are generally considered older and with more comorbidities. Additionally, patients undergoing PFR due to non-oncologic reasons tend to have an impaired soft-tissue envelope due to subsequent revision surgery. (Fig. 7)

**Table 4:** Proximal femoral reconstruction in a review of literature. Number of cases includes all patients analyzed in statistical considerations

Author	Journal	Year	Indication	Fixation	No. cases	Observational period	Implant survival
Malkani et al	JBJS Br	1995	Periprosthetic fracture Aseptic loosening Salvage of Girdlestone Fracture Conversion of arthrodesis	Cemented	30	11.1 (5.1–18.1) years	12 years: 64%
Haentjens et al	Acta Orthop Scand	1996	Aseptic loosening	Cemented	16	5 (2–11) years	No survival analysis
Klein et al	JBJS Am	2005	Periprosthetic fracture	Cemented	21	3.2 (2–7) years	No survival analysis
Parvizi et al	JBJS Am	2007	Periprosthetic Fracture PJI Aseptic loosening Non-union Fracture	Cemented	43	36.5 (24–79) months	1 year: 87% 5 years: 73%
Shih et al	Chang Gung Med J	2007	Infection or Fracture after Allograft-prosthesis composite Periprosthetic fracture Aseptic loosening PJI	Cemented	12	5.7 (3.3–9) years	5 years: 86.1% 10 years: 62.4%
Hardes et al	Z Orthop Unfall	2009	Periprosthetic fracture PJI Implant-associated infection Aseptic loosening	No information	28	46 (3–132) months	5 year: 81.8%
Al-Taki et al	Clin Orthop Relat Res	2011	Periprosthetic fracture Aseptic loosening PJI Recurrent dislocation	Mixed (33 Cemented, 3 Press fit)	36	3.2 (2–10) years	No survival analysis
Colman et al	J Arthroplasty	2014	Periprosthetic fracture	No information	21	15.2 months	1 year: 94.4% 5 years: 31.5% (Competing risk analysis)
Curtin et al	J Orthop	2017	Periprosthetic fracture	Mixed (14 Cemented, 2 Press fit)	16	19.2 (9–26) months	No survival analysis
Viste et al	Bone Joint J	2017	Periprosthetic fracture Aseptic loosening PJI Instability	Cemented	44	6 (2–12) years	5 years: 86% 10 years: 66%
Khajuria et al	Hip Int	2018	Loss of fracture reduction Periprosthetic fracture Aseptic loosening PJI Paediatric arthrodesis	Cemented	37	33 (6–84) months	1 year: 97.3% 5 years: 94.6%
De Martino et al	Int Orthop	2019	Periprosthetic fracture PJI Aseptic loosening Non-union	Mixed	31	5 (2–10) years	5 year: 78%
Fenelon et al	J Arthroplasty	2020	Periprosthetic fracture PJI Aseptic loosening Failed osteosynthesis Severe osteoarthritis Fracture	Cemented	79	31 (0–90) months	1 year: 96.2% 5 years: 94.9%
Döring et al	Int Orthop	2021	Periprosthetic fracture Aseptic loosening PJI Recurrent dislocation Fracture	Mixed (12 Cemented, 16 Press fit)	28	7.3 (1–25) years	1 year: 67.9% 5 years: 45.8% 10 years: 38.1%

### Complication-specific implant survival rates according to the ISOLS classification

To our knowledge, this study is the first to investigate the revision-free survival distinguished by failure mode. Only one research group has previously attempted to classify complications of megaprostheses in non-oncology settings [26]. The most frequent post-PFR complication observed among our patients was dislocation. Wide-ranging joint dislocation rates from 18 to 50% have been described in literature [22, 27–30]. Various factors contributing to dislocation have been proposed in preceding studies, some of which would help explain this leading complication of PFR in this study, such as advanced patient age at surgery and positive history for multiple surgeries of the proximal femur or hip [27, 30, 31]. In fact, multiple revision surgery is thought to lead to enlarged soft-tissue scars and lessened or inadequate abductor muscle tension that contribute to joint dislocation [27]. Several intra-operative strategies for dealing with PFR-accompanying dislocation have been proposed, such as acetabular cup positioning with a less than 30° lateral opening angle, abduction apparatus reattachment, deliberate limb lengthening as well as dual mobility constructs or constrained liners [10, 27]. Yet, post-operative care might very well be the most crucial element leading to improved hip stability. Most authors advise immobilization of the operated limb in abduction for various post-operative durations, and protected weight-bearing thereafter [22, 32–35]. Abduction bracing on mobilization appears to be the preferred method of dislocation prevention applied by orthopaedic surgeons [27, 30]. Surprisingly, patients immobilized with a cast or orthosis had a higher dislocation risk in this study (p = 0.01), with all dislocations occurring after immobilization with a cast or orthosis. We then further analyzed the cast and crutches only groups and found no statistical differences in patient age, modular reconstruction length, number of previous surgeries or indication for megaprosthesis implantation. We further found no differences in dislocation numbers when separating the index surgery date in two timeframes at the median, one before 2003 and one after 2003. We think that in our case series, casts or orthoses were used when the surgeon primarily suspected a high dislocation risk, which prove true in a retrospective view. With limited intra-operative options to account for that risk in the study inclusion period, surgeons were forced to restrict hip movement. We think that in modern revision proximal femoral reconstruction, the use of dual mobility liners or constrained liners should have a high impact in further lowering the dislocation risk. Even though dislocation comes across as the number one complication of salvage hip surgery, fracture, infection and limb length discrepancy are also leading post-surgical complications [7, 16].

In this study, a high rate of infection of megaprostheses and surrounding tissue was observed. However, a review of the literature revealed various authors that described comparable incidences of PJI between 6.5 and 16% following PFR [22, 27, 28, 30]. In comparison with patients who have undergone primary joint replacement, revision PFR seems to come with a high risk of PJI [36]. Furthermore, the risk for PJI seems to increase substantially in patients with positive history for infections of previously implanted prosthetic devices [30]. This was well-reflected in our own results, as 5 out of 6 patients with type IV complications had recurrent infections. Hence, megaprosthesis implantation in infection-eradicated tissue (after one- or two-stage exchange in patients diagnosed with PJI) [37, 38] did not reduce the relative risk for developing PJI and was still notably higher when compared to the relative risk for developing PJI in patients who did not suffer from previous PJI. In descending order of incidence, other complications of megaprostheses detected in our study population were periprosthetic fracture and aseptic loosening. Assessment of survival sorted by gender, age, previous surgery and previous infections revealed no preference for any one subgroup of patients. Also, no significant differences were found with a Cox regression model [39].

### PFR cementation as protective factor in implant survival

Only cemented PFR stems yielded superior results in comparison to press-fit implantation. Especially aseptic loosening was not recorded after PFR cementation. In this case series, a higher primary stability of cemented PFRs seemed to outweigh potential benefits of press-fit implantations in patients of impaired femoral bone and soft tissue quality due to multiple revision surgery and, again, a high number of infections prior to PFR implantation. In literature, some authors presented their results after revision PFR based on cemented prostheses only [8, 10, 40]. Thus, in contrast to young bone tumour patients with optimal bone quality and a potential higher demand, PFR cementation can be cautiously advocated in revision arthroplasty settings based on these studies’ results. However, larger comparative studies are needed for a definitive recommendation.

## Conclusion

PFR with special segmental bone and joint replacement systems in non-oncological patients is a complex surgical procedure that is associated with shortened implant survival and presents substantial risk for complications–dislocation, PJI, periprosthetic fracture and aseptic loosening. In patients with severe bone loss and very poor bone quality, salvage hip surgery with modular proximal femoral endoprostheses should only be used as a last resort. In these cases, PFR stem cementation should be the recommended option.

## Data Availability

Not applicable.

## References

[1] Kurtz S, Ong K, Lau E, Mowat F, Halpern M (2007). Projections of primary and revision hip and knee arthroplasty in the United States from 2005 to 2030. J Bone Joint Surg Am.

[2] Ingham E, Fisher J (2000). Biological reactions to wear debris in total joint replacement. Proc Inst Mech Eng H.

[3] Tanzer M, Kantor S, Rosenthall L, Bobyn JD (2001). Femoral remodeling after porous-coated total hip arthroplasty with and without hydroxyapatite-tricalcium phosphate coating: a prospective randomized trial. J Arthroplasty.

[4] Springer BD, Berry DJ, Lewallen DG (2003). Treatment of periprosthetic femoral fractures following total hip arthroplasty with femoral component revision. J Bone Joint Surg Am.

[5] Zaki SH, Sadiq S, Purbach B, Wroblewski BM (2007). Periprosthetic femoral fractures treated with a modular distally cemented stem. J Orthop Surg (Hong Kong).

[6] Klein GR, Parvizi J, Rapuri V, Wolf CF, Hozack WJ, Sharkey PF, Purtill JJ (2005). Proximal femoral replacement for the treatment of periprosthetic fractures. J Bone Joint Surg Am.

[7] Parvizi J, Sim FH (2004). Proximal femoral replacements with megaprostheses. Clin Orthop Relat Res.

[8] Parvizi J, Tarity TD, Slenker N, Wade F, Trappler R, Hozack WJ, Sim FH (2007). Proximal femoral replacement in patients with non-neoplastic conditions. J Bone Joint Surg Am.

[9] Rasouli MR, Porat MD, Hozack WJ, Parvizi J (2012). Proximal femoral replacement and allograft prosthesis composite in the treatment of periprosthetic fractures with significant proximal bone loss. Orthop Surg.

[10] Viste A, Perry KI, Taunton MJ, Hanssen AD, Abdel MP (2017). Proximal femoral replacement in contemporary revision total hip arthroplasty for severe femoral bone loss: a review of outcomes. Bone Joint J.

[11] Savvidou OD, Mavrogenis AF, Sakellariou V, Christogiannis I, Vottis C, Christodoulou M, Vlasis K, Papagelopoulos PJ (2014). Salvage of failed total hip arthroplasty with proximal femoral replacement. Orthopedics.

[12] Al-Taki MM, Masri BA, Duncan CP, Garbuz DS (2011). Quality of life following proximal femoral replacement using a modular system in revision THA. Clin Orthop Relat Res.

[13] Gkavardina A, Tasgozis P (2014). The use of megaprostheses for reconstruction of large skeletal defects in the extremities: a critical review. Open Orthop J.

[14] Lundh F, Sayed-Noor AS, Brosjo O, Bauer H (2014). Megaprosthetic reconstruction for periprosthetic or highly comminuted fractures of the hip and knee. Eur J Orthop Surg Traumatol.

[15] Giannoudis PV (2016). Treatment of bone defects: bone transport or the induced membrane technique?. Injury.

[16] Korim MT, Esler CN, Ashford RU (2014). Systematic review of proximal femoral arthroplasty for non-neoplastic conditions. J Arthroplasty.

[17] Potter BK, Chow VE, Adams SC, Letson GD, Temple HT (2009). Endoprosthetic proximal femur replacement: metastatic versus primary tumors. Surg Oncol.

[18] Menendez LR, Ahlmann ER, Kermani C, Gotha H (2006). Endoprosthetic reconstruction for neoplasms of the proximal femur. Clin Orthop Relat Res.

[19] Chandrasekar CR, Grimer RJ, Carter SR, Tillman RM, Abudu A, Buckley L (2009). Modular endoprosthetic replacement for tumours of the proximal femur. J Bone Joint Surg Br.

[20] Kotz R (1993). Tumor endoprosthesis in malignant bone tumors. Orthopade.

[21] Henderson ER, Groundland JS, Pala E, Dennis JA, Wooten R, Cheong D, Windhager R, Kotz RI, Mercuri M, Funovics PT, Hornicek FJ, Temple HT, Ruggieri P, Letson GD (2011). Failure mode classification for tumor endoprostheses: retrospective review of five institutions and a literature review. J Bone Joint Surg Am.

[22] Malkani AL, Settecerri JJ, Sim FH, Chao EY, Wallrichs SL (1995). Long-term results of proximal femoral replacement for non-neoplastic disorders. J Bone Joint Surg Br.

[23] Sewell MD, Hanna SA, Carrington RW, Pollock RC, Skinner JA, Cannon SR, Briggs TW (2010). Modular proximal femoral replacement in salvage hip surgery for non-neoplastic conditions. Acta Orthop Belg.

[24] Colman M, Choi L, Chen A, Crossett L, Tarkin I, McGough R (2014). Proximal femoral replacement in the management of acute periprosthetic fractures of the hip: a competing risks survival analysis. J Arthroplasty.

[25] Jameson SS, Kyle J, Baker PN, Mason J, Deehan DJ, McMurtry IA, Reed MR (2012). Patient and implant survival following 4323 total hip replacements for acute femoral neck fracture: a retrospective cohort study using National Joint Registry data. J Bone Joint Surg Br.

[26] De Martino I, D’Apolito R, Nocon AA, Sculco TP, Sculco PK, Bostrom MP (2019). Proximal femoral replacement in non-oncologic patients undergoing revision total hip arthroplasty. Int Orthop.

[27] Haentjens P, De Boeck H, Opdecam P (1996). Proximal femoral replacement prosthesis for salvage of failed hip arthroplasty: complications in a 2–11 year follow-up study in 19 elderly patients. Acta Orthop Scand.

[28] Johnsson R, Carlsson A, Kisch K, Moritz U, Zetterstrom R, Persson BM (1985). Function following mega total hip arthroplasty compared with conventional total hip arthroplasty and healthy matched controls. Clin Orthop Relat Res.

[29] Zehr RJ, Enneking WF, Scarborough MT (1996). Allograft-prosthesis composite versus megaprosthesis in proximal femoral reconstruction. Clin Orthop Relat Res.

[30] Shih ST, Wang JW, Hsu CC (2007). Proximal femoral megaprosthesis for failed total hip arthroplasty. Chang Gung Med J.

[31] Newington DP, Bannister GC, Fordyce M (1990). Primary total hip replacement in patients over 80 years of age. J Bone Joint Surg Br.

[32] Bosquet M, Burssens A, Mulier JC (1980). Long term follow-up results of a femoral megaprosthesis. A review of thirteen patients. Arch Orthop Trauma Surg.

[33] Katzner M, Jacquemaire B, Babin S, Schvingt E (1979). Total hip arthroplasties with special long prothesis following resection of the upper femoral shaft 62 cases. Technic, indications and results (author's transl). Ann Chir.

[34] Katzner M, Schvingt E (1982). Study of 100 total arthroplasties of the hip with femoral megaprosthesis after extensive resection of the upper end of the femur. Int Orthop.

[35] Sim FH, Chao EY (1981). Hip salvage by proximal femoral replacement. J Bone Joint Surg Am.

[36] Darouiche RO (2004). Treatment of infections associated with surgical implants. N Engl J Med.

[37] Kunutsor SK (2018). One- and two-stage surgical revision of peri-prosthetic joint infection of the hip: a pooled individual participant data analysis of 44 cohort studies. Eur J Epidemiol.

[38] Leite PS, Figueiredo S, Sousa R (2016). Prosthetic joint infection: report on the one versus two-stage exchange EBJIS survey. J Bone Jt Infect.

[39] Kocak M, Onar-Thomas A (2012). A simulation based evaluation of the asymptotic power formulae for cox models in small sample cases. Am Stat.

[40] Fenelon C, Murphy EP, Kearns SR, Curtin W, Murphy CG (2020). Cemented Proximal Femoral Replacement for the Management of Non-Neoplastic Conditions: A Versatile Implant but Not Without Its Risks. J Arthroplasty.

